# Improving *In Vitro* Generated Cartilage-Carrier-Constructs by Optimizing Growth Factor Combination

**DOI:** 10.2174/1874120700701010085

**Published:** 2007-12-13

**Authors:** Katharina Wiegandt, Christiane Goepfert, Ralf Pörtner

**Affiliations:** Bioprocess and Biosystems Engineering, Hamburg University of Technology, Germany

**Keywords:** Cartilage, osteochondral implants, growth factors, IGF-I, TGF-β1, re-differentiation.

## Abstract

The presented study is focused on the generation of osteochondral implants for cartilage repair, which consist of bone substitutes covered with *in vitro* engineered cartilage. Re-differentiation of expanded porcine cells was performed in alginate gel followed by cartilage formation in high-density cell cultures. In this work, different combinations of growth factors for the stimulation of re-differentiation and cartilage formation have been tested to improve the quality of osteochondral implants. It has been demonstrated that supplementation of the medium with growth factors has significant effects on the properties of the matrix. The addition of the growth factors IGF-I (100 ng/mL) and TGF-β1 (10 ng/mL) during the alginate culture and the absence of any growth factors during the high-density cell culture led to significantly higher GAG to DNA ratios and Young’s Moduli of the constructs compared to other combinations. The histological sections showed homogenous tissue and intensive staining for collagen type II.

## INTRODUCTION

Full-thickness cartilage defects of the knee joint lead to pain and restriction in movement. Its treatment is still a common problem as existing therapies for the repair of articular cartilage injuries do not offer complete restoration [1, 2].

Methods of tissue engineering have opened up new strategies for the re-construction of cartilage. During the last years several approaches in cartilage tissue engineering have shown promising but not satisfying results which motivate for further studies [1-3]. In this work, osteochondral implants which consist of a ceramic carrier as bone substitute and a layer of cultivated cartilage are generated *in vitro*. A mosaic-like implantation [2, 4] of these autologous cartilage-carrier-constructs may provide a reconstructed surface area inside the knee joint. The cultivation principle includes the following steps [5, 6]: (a) explanted chondrocytes are expanded in monolayer culture until passage 3; (b) afterwards the cells are seeded onto a solid carrier (cell coating) and cultivated for two weeks; (c) simultaneously expanded chondrocytes are suspended in alginate gel for two weeks to induce re-differentiation; (d) the re-differentiated chondrocytes are then eluted out of the alginate gel and sedimented on the cell coated carrier. These cartilage-carrier-constructs are cultivated for cartilage formation for three weeks (high-density cell culture). The concept was applied successfully in mini-pigs [7].

The initial cell number for the generation of autologous implants is limited by the small size of a biopsy which is assumed to count 0.2x10^6^ to 1.2x10^6^ cells. Hence, chondrocytes have to be expanded until the required cell number is reached; for example, for cartilage-carrier-constructs as per the presented method, with an initial cell number of 1x10^6^ cells it is necessary to subcultivate monolayer cultures until passage 3 in order to fill an area of approximately 6 cm^2^ in the joint. But, proliferation is accompanied by dedifferentiation of cells. De-differentiated chondrocytes stop production of cartilage-specific extracellular matrix components, especially collagen type II and glycosaminoglycans, and acquire fibroblast-like morphology [8, 9]. To undergo chondrogenesis, cells have to re-differentiate, which is still under investigation *in vitro*. Re-differentiation can be supported by the addition of certain growth factors to the culture medium [9-11].

Research being carried out to determine the characteristics and effects of growth factors in cartilage tissue engineering has proved that IGF-I (Insulin-like Growth Factor I) especially and TGF-β1 (Transforming Growth Factor β1) delivered positive results. Both growth factors, IGF-I and TGF-β1, can be produced by chondrocytes and are accumulated in the extracellular matrix as a complex with binding proteins (IGF-I) or in a latent form (TGF-β1) respectively [12]. *In vivo, *for instance, IGF-I expression is increased during healing of full-thickness cartilage defects [13] and TGF-β1 is present in tissues during differentiation [14]. From several studies, it is known that IGF-I and TGF-β1 influence differentiation of chondrocytes and cartilage formation *in vitro *as well. The varying results concerning the effects of these growth factors are attributed to the differences in culture conditions, species and age of donors, growth factor concentrations, status of cell differentiation or the addition of serum [15, 16]. To summarize, IGF-I is an important anabolic factor for chondrocytes and *in vitro* engineered cartilage. It can stimulate the proliferation of cells and extracellular matrix biosynthesis, in particular the production of proteoglycans [8, 11, 16, 17]. The addition of TGF-β1 to the culture medium has shown diverse effects on the cultivation of chondrocytes. It can stimulate or inhibit the cell growth and accumulation of proteoglycans and collagen type II [11]. Tsukazaki *et al. *observed that TGF-β1 acts synergistically with IGF-I, which means that a combination of both growth factors can influence the cultivation in a different way than can each growth factor alone [12, 16].

Thus, growth factor supplements can improve the quality of engineered cartilage, but they have to be adjusted for each application in tissue engineering. In the current study, the effects of IGF-I and TGF-β1 were examined during the redifferentiation in alginate beads (step c) and cartilage formation in high-density cell cultures (step d) for the cultivation principle introduced above in order to optimize the biochemical and biomechanical properties of the cartilage-carrier-constructs *in vitro.* Porcine chondrocytes were used as an animal model system as animal experiments have to precede later clinical applications.

## MATERIALS AND METHODS

### Culture Procedure for Generation of Cartilage-Carrier-Constructs

Porcine chrondrocytes were isolated from a knee joint of an approximately 5 month old domestic pig. Small pieces of cartilage were taken from femur and digested with hyaluronidase type III-solution (0.5 mg/mL, Sigma-Aldich, Germany) and with trypsin/EDTA-solution (PAA, Germany). The cartilage was further treated with collagenase type Ia-solution (0.5 mg/mL, Sigma-Aldrich) overnight.

Cells were cultured in T-flasks (Roth, Germany) until passage 3. The initial cell number was 2x10^5^ cells for the generation of six constructs. Proliferation of the cells was performed in DMEM (Dulbecco`s Modified Eagle Medium, PAA) supplemented with 10% (v/v) fetal calf serum (FCS, PAA), penicillin/streptomycin (100 U/mL penicillin and 100 µg/mL streptomycin, PAA) and 10 ng/mL human basic Fibroblast Growth Factor (bFGF, CellConcepts, Germany) [18].

Cartilage-carrier-constructs were generated according to the concept described above [6]. Briefly, after harvesting cells from the T-flasks, 2x10^5 ^chondrocytes were sedimented onto a calcium-phosphate-carrier (diameter 4.55 mm, Sponceram HA^®^, Zellwerk, Germany) to form a cell layer. During the cultivation of the cell layer, the above mentioned medium was used to stimulate cell proliferation [18].

In parallel, chondrocytes from the same preculture were encapsulated in alginate beads (1x10^6^ cells per mL alginate, Sigma-Aldrich) and cultivated for two weeks. Afterwards, the cells were recovered from the gel by using citrate buffer and centrifuged onto the cell coated carrier (1.8x10^6^ cells per carrier).

The cartilage-carrier-constructs were then cultivated for three weeks in a high-density cell culture. During the redifferentiation in alginate beads and the following cartilage formation of the cartilage-carrier-constructs, DMEM (PAA) supplemented with 10% (v/v) porcine serum (PS, Gibco, Germany), penicillin/streptomycin (100 U/mL penicillin and 100 µg/mL streptomycin, PAA), 0.28 mM L-ascorbic acid 2-phosphate and 1 mM cysteine (Sigma-Aldrich) was used. Furthermore, different growth factors were added to the medium. The medium was exchanged three times a week.

All cultivations were performed under an atmosphere of 5% (v/v) O_2_ and 5% (v/v) CO_2_.

### Variation of Growth Factor Combination

To support re-differentiation in alginate gel and cartilage formation of cartilage-carrier-constructs, different growth factor combinations were tested. hIGF-I (100 ng/mL human IGF-I, Cell Concepts, Germany) or/and hTGF-β1 (10 ng/mL human TGF-β1, Cell Concepts) were added to the medium [Goepfert, unpublished].

In the alginate gel, the cells were cultured either without any growth factors, with hIGF-I or with hIGF-I and hTGF-β1 at the same time. hTGF-β1 alone was not used as preliminary tests showed a strong decrease in the production of cartilage-specific matrix components like collagen type II and glycosaminoglycans (unpublished data of the authors). Afterwards, during the cartilage formation, the cartilage-carrier-constructs were cultivated with hIGF-I or without any growth factors. The different combinations are listed in Table **1**. For each combination, six constructs were prepared.

### Biochemical Analysis

To analyze biochemical properties of the extracellular matrix, glycosaminoglycan (GAG) content and amount of DNA were determined. The method is based on a work by Buschmann et al. [19]. Cartilage tissue was digested enzymatically with papain (Roche, Germany). The GAG content was then determined by staining with 1,9-dimethylene blue chloride (Serva, Germany) with chondroitin sulfate (Sigma-Aldrich) as the standard and the DNA content using the fluorescence marker H33258 (Sanofi-Aventis, Germany) with calf thymus DNA as the standard (for details see [5, 6]).

### Immunochemical Analysis

To determine the state of re-differentiation of cells after alginate culture, the recovered chondrocytes were centrifuged onto object slides (Histo-Bond, Marienfeldt, Germany) and then immunostained against collagen type I and collagen type II [20]. In the first step, monoclonal antibodies of mice (Acris Antibodies, Germany) were found to bind specifically to collagen type I (clone I-8H5) or collagen type II (clone II-4C11). Afterwards, a secondary fluorescent antibody (Goat anti-mouse, [IgG (H+L), FITC], Southern Biotech, USA) was used for staining. The staining of the nuclei was performed with DNA-binding fluorochrome 4-6-diamidino-2-phenyl-di-hydro-chloride (Dapi, Sigma-Aldrich). The total amount of cells and the amount of cells generating collagen were analyzed using a fluorescence microscope (Nikon, Germany) and the corresponding software NIS Elements AR.

To determine the collagen type I and II formation of cartilage-carrier-constructs, the generated tissue was embedded in paraffin and 5 µm thick histological sections were prepared with a microtome (Leica, Germany). The histological sections were immunostained separately with primary antibodies (Acris Antibodies) against collagen type I and collagen type II. As the secondary antibody, a biotinylated antibody (Goat anti-mouse, [IgG (H+L)-biotin], Southern Biotech) was used. Color development was carried out using strepatividin/alkaline phosphatase complex (Linaris, Germany) and the New Fuchsin chromogen (Sigma-Aldrich) [20]. Nuclei were stained with haematoxilin (Roth).

### Biomechanical Parameters

At the end of each cultivation, the wet weight (Mettler AE200, Germany), the height and the Young’s Modulus of the cartilage-constructs were measured. For determination of the height and the Young’s Modulus, a high-precision material testing machine (Zwicki 1120, Zwick, Germany) was used. For the determination of the Young’s Modulus stepwise stress-relaxation tests (five steps with 4% of the uncompressed cartilage thickness each) were carried out according to Korhonen *et al.* [21]. The criterion for relaxation was a relaxation rate of less than 0.002 N/min. The Young’s Modulus was calculated from resulting stress-strain curve.

### Statistics

Statistics software NCSS97 was applied to evaluate statistical significance of the data (p < 0.05, ANOVA).

## RESULTS

### Alginate Culture

In order to evaluate re-differentiation after recovering cells from the alginate gel, the GAG to DNA ratio and the percentage of collagen type I and collagen type II producing cells were determined.

Fig. (**1**) shows that the absence of any growth factors during the alginate culture resulted in fewer collagen type I and collagen type II producing cells compared to the other supplementations. Not only the percentage of collagen type II producing cells in the cultures using IGF-I or IGF-I and TGF-β1 rose to nearly 100%, but also an increase of collagen type I could be determined.

The significantly lowest GAG to DNA ratios were found during the cultivation with IGF-I and TGF-β1. Compared to this, the other conditions led to relatively high GAG to DNA ratios of more than 40 µg/µg.

### Cartilage-Carrier-Constructs

The absence of any growth factors (w/o GF) during the alginate culture led to irregularly shaped cartilage-carrier-constructs (Fig. **2/1, 2/2**). Using only IGF-I during cultivation in alginate beads resulted in very soft and unstable cartilage. In Fig. **2**, it is visible that these constructs (Fig. **2/3, 2/4**) consist of only small amounts of tissue on top of the carrier. Because of the low height of these constructs a biochemical and biomechanical analysis was not possible and only the other combinations were considered for further studies (Fig. **2/1, 2/2, 2/5, 2/6**).

The largest constructs were found for the supplementation with IGF-I and TGF-β1 during the alginate culture and IGF-I during the cartilage formation. These observations were also confirmed by the data obtained for the wet weight and height of the engineered cartilage presented (data in Fig. **3**). Comparing the biomechanical parameters showed that the constructs cultivated with IGF-I and TGF-β1 during the alginate culture and without any growth factors during the cartilage formation exhibited the significantly highest Young’s Modulus of 0.0595 MPa.

The DNA concentration of cartilage-carrier-constructs cultured without any growth factors during cartilage formation was nearly twice as high as those using growth factors (Fig. **4**). However, the GAG fraction of constructs is a more relevant factor for evaluation of cartilage as it influences the biomechanical properties of tissue [22]. It increased when IGF-I and TGF-β1 were used during the alginate culture. Fig. **4** shows the significantly highest GAG to DNA ratio of 116 µg/µg for the constructs cultivated with IGF-I and TGF-β1 during alginate culture and with IGF-I during cartilage formation.

The immunohistological images (Fig. **5**) show a more prominent staining for collagen type II than for collagen type I in all cases. When cultivations were performed without any growth factors during the alginate culture, the constructs showed a tissue interspersed with holes. In contrast, the cultivation with IGF-I and TGF-β1 resulted in homogenous tissue in which chondrocytes were evenly distributed.

## DISCUSSION

One approach for the treatment of full-thickness cartilage defects is the generation of autologous osteochondral implants with the aid of tissue engineering methods. To minimize the donor site morbidity due to a biopsy, it is necessary to expand the chondrocytes [8], which leads to a de-differentiation of the cells. It is possible to induce re-differentiation by three-dimensional cultivation and the addition of growth factors. Many studies have demonstrated positive effects of the growth factors IGF-I and TGF-β1 [9-11, 16, 17]. The present work was designed to determine an optimal combination of growth factor supplementation for our cultivation principle and thereby to improve the cartilage formation. Therefore, the expanded cells were first re-differentiated in an alginate gel and then further cultivated as cartilage-carrier-constructs for three weeks. Different combinations (Table **1**) of the growth factors IGF-I and TGF-β1 were tested during the re-differentiation in alginate beads and afterwards during the cartilage formation of the constructs.

In this work, it is shown that it is not suitable to investigate the phases of the cultivation principle separately as the different steps are influenced by each other. Furthermore, it can be concluded that chondrocytes react in a different way to certain growth factors during re-differentiation or cartilage formation.

The absence of any growth factors during the cultivation in alginate gel resulted in a high GAG to DNA ratio of 40.1 µg/µg, but the number of cells which produced collagen type II were fewer than the cells cultured with IGF-I or IGF-I and TGF-β1. Collagen type II is an important extracellular matrix component, while collagen type I is generally not present in articular cartilage [8]. Hence, these results demonstrate that the investigated growth factors can support the re-differentiation, here identified by the collagen type II production [9-11, 16, 17]. The generated constructs using no growth factors during alginate culture showed an irregular shape and tissue interspersed with holes.

**Fig. (1):**
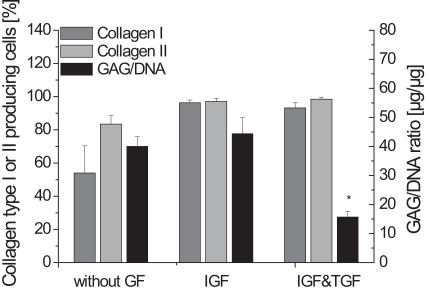
Biochemical parameters GAG to DNA ratio and collagen type I and collagen type II producing cells of the alginate culture, either without any growth factors, with IGF-I or IGF-I and TGF-β1 (n=4, standard deviation of the mean, * significantly lowest GAG to DNA ratio).

**Fig. (2):**
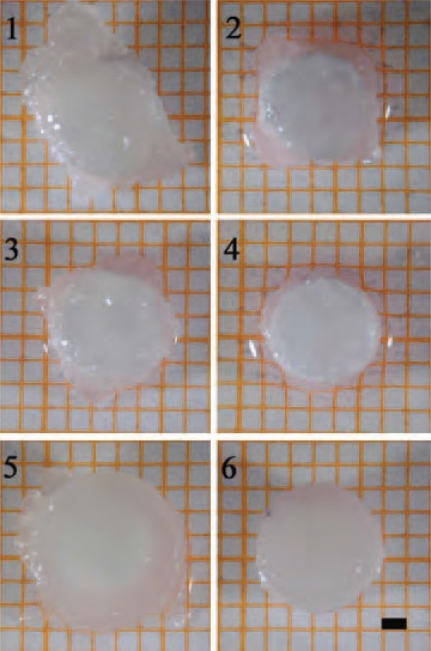
Top view of cartilage-carrier-constructs (1) alginate gel: w/o GF, cartilage formation: IGF-I; (2) alginate gel: w/o GF, cartilage formation: w/o GF; (3) alginate gel: IGF-I, cartilage formation: IGF-I; (4) alginate gel: IGF-I, cartilage formation: w/o GF; (5) alginate gel: IGF-I&TGF-β1, cartilage formation: IGF-I; (6) alginate gel: IGF-I&TGF-β1, cartilage formation: w/o GF; (scale bar= 1 mm).

**Fig. (3):**
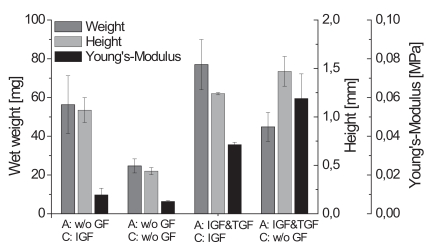
Biomechanical parameters weight, height and Young’s Modulus of cartilage-carrier-constructs cultivated with different growth factor combinations (A - alginate culture, C - cartilage formation; n=6, standard deviation of the mean, significances not shown).

**Fig. (4):**
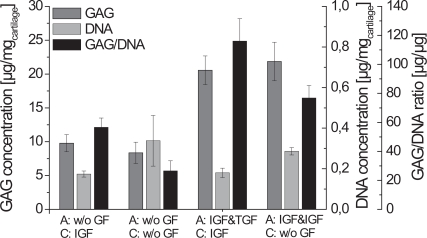
Relationship between GAG- and DNA-content of cartilage-carrier-constructs cultivated with different growth factor combinations (A - alginate culture, C -cartilage formation; n=6, standard deviation of the mean, significances not shown).

**Fig. (5):**
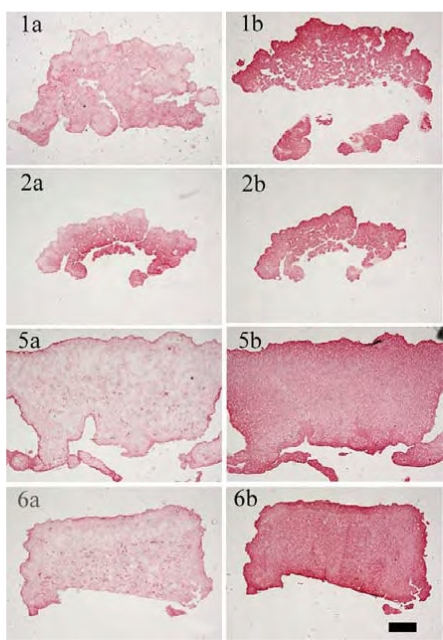
Immunohistological staining: left column for collagen type I, right column for collagen type II (1a)/(1b) alginate gel: w/o GF, cartilage formation: IGF-I; (2a)/(2b) alginate gel: w/o GF, cartilage formation: w/o GF; (5a)/(5b) alginate gel: IGF-I&TGF-β1, cartilage formation: IGF-I; (6a)/(6b) alginate gel: IGF-I&TGF-β1, cartilage formation: w/o GF; condition 3 and 4 not shown (scale bar= 100 µm)

The analysis of the alginate cultures led to the assumption that cells cultivated with IGF-I result in constructs with improved matrix properties compared to the cultivation without any growth factors or with IGF-I and TGF-β1. Fig. **1** shows a GAG to DNA ratio of 44.4 µg/µg and a collagen type II to I ratio of 1.06. Nearly 100 % of the cells produced the cartilage-specific collagen type II, but unfortunately also collagen type I. However, the resulting tissue was soft and no stable cartilage-carrier-constructs could be prepared from it. It is assumed that the cells have been stimulated by IGF-I to produce large amounts of proteoglycans. This presumption corresponds to the findings of many other groups [8, 11, 12]. But possibly, the collagen network did not develop adequately to maintain the cells as a pellet. These data demonstrate that the production of large amounts of proteoglycans during the alginate culture does not necessarily lead to the formation of high quality cartilage. Thus, addition of IGF-I during the alginate culture is not sufficient for our application.

The GAG to DNA ratio after the alginate culture using IGF-I and TGF-β1 was only 15.7 µg/µg which is significantly lower than the other combinations. During their studies, Blunk *et al. *[11] also found, compared to the standard conditions, a decrease of the glycosaminoglycan fraction, when bovine calf chondrocytes were cultivated with IGF-I and TGF-β1 on polyglycolic acid scaffolds. Nevertheless, when using IGF-I and TGF-β1 during the alginate culture resulted in constructs with the highest biochemical and biomechanical properties compared to the other conditions in our work.

Table **2** shows a summary of the biochemical and biomechanical parameters of the generated cartilage-carrier-constructs. Constructs which were cultivated first with IGF-I and TGF-β1 and afterwards during the cartilage formation with IGF-I yielded the significantly highest GAG to DNA ratio of 116.1 µg/µg, which is already 70 % of that of native cartilage. In contrast, the significantly highest Young’s Modulus was observed for the constructs which were cultivated with IGF-I and TGF-β1 during the re-differentiation in alginate beads and without any growth factors during the cartilage formation. The Young’s Modulus achieved (0.0595 MPa) was only 15 % of that of native cartilage. For both conditions, histological sections showed homogenous tissue with an intensive staining against collagen type II. According to the presented results, the biochemical appearance of the tissue engineered cartilage is close to native cartilage, while it is still necessary to improve the biomechanical properties.

Articular cartilage *in vivo* has to transmit high stress [13]. Thus, it is important that tissue engineered cartilage can withstand these loadings. Because the Young’s Modulus is an indicator for the stiffness of the extracellular matrix, we decided to proceed with our research using IGF-I and TGF-β1 as medium supplements during the re-differentiation in alginate beads and no growth factors during the cartilage formation in high-density cell cultures. During cultivation *in vitro* biomechanical parameters may be increased by mechanical load like hydrostatic pressure, direct compression or shear stress. From several studies it is known that these mechanical stimuli influence the production and organization of cartilage-specific matrix components [23, 24].

## CONCLUSION

In this study, it was shown that the addition of certain growth factors during the generation of osteochondral implants had significant effects on the properties of the matrix. We were able to determine the required optimal growth factor combinations during the different steps of the presented cultivation scheme, which support the re-differentiation and cartilage formation *in vitro *and thereby improve the biomechanical and biochemical properties of porcine cartilage-carrier-constructs. It is still required to further increase fractions of extracellular matrix, especially collagen type II, and to improve the biophysical parameters. In addition, it is needed to investigate an effective growth factor combination for adult human chondrocytes.

## Figures and Tables

**Table 1:** Experimental Set-Up

No.	Alginate Gel	Cartilage Formation
1.	Without growth factors	100 ng/mL hIGF-I
2.		Without growth factors
3.	100 ng/mL hIGF-I	100 ng/mL hIGF-I
4.		Without growth factors
5.	100 ng/mL hIGF-I & 10 ng/mL hTGF-β1	100 ng/mL hIGF-I
6.		Without growth factors

**Table 2:** Summary of biochemical and biomechanical parameters of the generated cartilage-carrier-constructs (1.) alginate gel: w/o GF, cartilage formation: IGF-I; (2.) alginate gel: w/o GF, cartilage formation: w/o GF; (5.) alginate gel: IGF-I &TGF-β1, cartilage formation: IGF-I; (6.) alginate gel: IGF-I &TGF-β1, cartilage formation: w/o GF; condition 3. and 4. not shown

Condition	1.	2.	5.	6.
Height [mm]	1.07±0.13	0.44±0.04	1.24±0.01	1.47±0.02
Wet weight [mg]	56.28±15.0	24.68±3.64	76.89±13.0	44.8±7.51
Young’s Modulus [MPa]	0.0098±0.003	0.0064±0.001	0.0357±0.001	0.0595±0.013
DNA [µg/mg_cartilage_]	0.174±0.015	0.338±0.125	0.180±0.023	0.286±0.018
GAG [µg/mg_cartilage_]	9.78±1.27	8.35±1.58	20.56±2.12	21.85±2.84
GAG/DNA [µg/µg]	56.62±6.63	26.57±6.97	116.09±15.43	76.88±8.57

## References

[1] Petersen J, Ruecker A, von Stechow D (2003). “Present and future therapies of articular cartilage defects”. Eur. J. Trauma.

[2] Behrens P, Bosch U, Bruns J (2004). “Recommendations for indication and application of ACT of the joint advisory board of the German Society for Traumatology (DGU) and Orthopaedic Surgery (DGOOC)”. Zeitschrift für Orthopädie und Unfallchirugie.

[3] Hunziher E (2001). “Articular cartilage repair: basic science and clinical progress. A review of the current status and prospects”. Osteoarthritis Cartilage.

[4] Hangody L, Füles P (2003). “Autologous osteochondral mosaicplasty for the treatment of full-thickness defects of weight-bearing joints”. J. Bone Joint Surg. Am. Vol.

[5] Nagel-Heyer S, Goepfert C, Morlock M, Pörtner R (2005). “Relationship between physical, biochemical and biomechanical properties of tissue-engineered cartilage-carrier-constructs”. Biotechnol. Lett.

[6] Nagel-Heyer S, Goepfert C, Feyerabend F (2005). “Bioreactor cultivation of three-dimensional cartilage-carrier-constructs”. Bioproc. Biosyst. Eng.

[7] Petersen J, Üblacker P, Goepfert C “Long term results after implantation of tissue engineered cartilage for the treatment of osteochondral lesions in a mini pig model”. J Mater Sci: Mater. Med.

[8] Pei M, Seidel J, Vunjak-Novakovic G, Freed L (2002). “Growth factors for sequential cellular de- and re-differentiation in tissue engineering”. Biochem. Biophys. Res. Commun.

[9] Jakob M, Demarteau O, Schäfer D (2001). “Specific growth factors during the expansion and redifferentiation of adult human articular chondrocytes enhances chondrogenesis and cartilaginous tissue formation”. J. Cell Biochem.

[10] Benz K, Breit S, Lukoschek M, Mau H, Richter W (2002). “Molecular analysis of expansion, differentiation, and growth factor treatment of human chondrocytes identifies differentiation markers and growth-related genes”. Biochem. Biophys. Res. Commun.

[11] Blunk T, Sieminski A, Gooch K (2002). “Differential effects of growth factors on tissue-engineered cartilage”. Tissue Eng.

[12] Tsukazaki T, Usa T, Matsumoto T (1994). “Effect of transforming growth factor-β on the insulin-like growth factor-I autocrine/paracrine axis in cultured rat articular chondrocytes”. Exp. Cell Res.

[13] Mauck R, Nicoll S, Seyhan S, Athesian G, Hung C (2003). “Synergistic action of growth factors and dynamic loading for articular cartilage tissue engineering”. Tissue Eng.

[14] Frenz D, Liu W, Williams J (1994). “Induction of chondrogenesis: requirement for synergistic interaction of basic fibroblast growth factor and transforming growth factor-beta”. Development.

[15] Guerne P, Sublet A, Lotz M (1994). “Growth factor responsiveness of human articular chondrocytes: distinct profiles in primary chondrocytes, subcultured chondrocytes, and fibroblasts”. J. Cell Physiol.

[16] Yaeger P, Masi T, Buck de Ortiz J (1997). “Synergistic action of transforming growth factor-ß and insulin like growth factor-I induces expression of type II collagen and aggrecan genes in adult human articular chondrocytes”. Exp. Cell Res.

[17] Van Osch G, Van den Berg W, Hunziker E, Häuselmann H (1998). “Differential effects of IGF-1 and TGF beta-2 on the assembly of proteoglycans in pericellular and territorial matrix by cultured bovine articular chondrocytes”. Osteoarthritis Cartilage.

[18] Martin I, Vunjak-Novakovic G, Yang J, Langer R, Freed L (1999). “Mammalian chondrocytes expanded in the presence of fibroblast growth factor 2 maintain the ability to differentiate and regenerate three-dimensional cartilaginous tissue”. Exp. Cell Res.

[19] Buschmann M, Gluzband Y, Grodzinski A, Kimura J, Hunziher E (1992). “Chondrocytes in agarose culture synthesis a mechanically functional extracellular matrix”. J. Orthopaed. Res.

[20] Goepfert C, Lutz V, Lünse S (2006). “Redifferentiation capacity of human articular chondrocytes (HAC) expanded on microcarriers”.

[21] Korhonen R, Laasanen M, Toyras J (2002). “Comparison of the equilibrium response of articular cartilage in unconfined compression, confined compression and indentation”. J. Biomechan.

[22] Heath C (2000). “The effect of physical forces on cartilage tissue engineering”. Biotechnol. Genet. Eng. Rev.

[23] Darling E, Athanasiou K (2003). “Review - Articular cartilage bioreactors and bioprocesses”. Tissue Eng.

[24] Heyland J, Wiegandt K, Goepfert C (2006). “Redifferentiation of chondrocytes and cartilage formation under intermittent hydrostatic pressure”. Biotechnol. Lett.

